# Discrimination and Dissatisfaction among Nurses Is a Threat for Objectives of Policies?

**Published:** 2020-04

**Authors:** Mojtaba MEHTARPOUR, Ebrahim JAAFARIPOOYAN

**Affiliations:** Department of Health Management and Economics, School of Public Health, Tehran University of Medical Sciences, Tehran, Iran

## Dear Editor-in-Chief

Upon the arrival of Rouhani’s government in 2013 and in line with his presidential election manifest, the Ministry of Health and Medical Education (MoHME) initiated a series of reforms, so-called Iran’s Health Transformation Plan (HTP), with three main goals: (a) promotion of financial protection; (b) increasing equity in access to health services; and (c) improving quality of health services. Accordingly, to meet the above mentioned, such interventions as reducing the out-of-pocket payments for inpatient services (from around previous 60%); improving the quality of medical and non-medical services; promoting the natural delivery; increasing the accessibility to specialists in deprived areas (through public hospitals), and updating of the relative value units (RVU) of tariffs were introduced. These measures initially increased highly the patients’ and fairly physicians’ satisfaction. However, the plan caused some discontent, particularly among the nurses (1). This dissatisfaction has presumably had some roots in the previous reforms and been to some extent intensified by the recent HTP, as they were complaining even before the HTP. Their grievances mainly ranged from discrimination in wages/salaries and job benefits and a high job burden (due to the inadequate nursing staff), resulting in domino-like consequences, such as fatigue and burnout, lack of knowledge and motivation, and severe stress (2).

Nurses strongly believed that after the HTP, they have been experiencing discrimination in payments in favor of physicians. In addition, extremely low costs services in public hospitals, following the HTP, have led to a heavy workload, intensifying their fatigue and dissatisfaction. There were besides complaints about an increasing violence against nurses, and declined prestige after HTP implementation (3).

In the third step of the HTP, RVUs were revised, in an effort to eliminate the informal payments and realize the values and tariffs of the service. The latter is argued to be the most important reason for the nurse’s dissatisfaction and alleged discrimination.

According to the street-level bureaucrats’ theory, nurses act as the ultimate implementers of healthcare policies and are those who translate them into practice. Nurses, as the street-level bureaucrats, communicate with citizens directly during providing, and in this process, they might influence the quantity and quality of health care services by their discretion, which lies in the nature of nursing jobs. Nurses could make decisions at the workplace that in a way might shape the organizations’ policies because citizens experience the final impact of any policy through the benefits enjoyed or losses faced by these bureaucrats(4). Therefore, nurses play a vital role in implementing the quality improvement of health care services. The question is whether nurses are trying to achieve the goals of an HTP intervention eagerly, while they are experiencing unhappiness with the implications of another. To realize the goals of health reform, relevant interventions must complement each other perfectly like puzzle pieces, suited to make a desired and perfect picture.

During policy implementation, factors such as the socio-political context, the working environment, personal beliefs and value systems can affect the behavior of nurses(4). If ignored, they may prevent intended goals. For effective implementation, at the policy formulating stage, a comprehensive approach needs to be employed to address all the practical aspects and prerequisites from policy practitioners - those who are able to change the objectives set out in policies and could make a policy failure.

## References

[1] Moradi-Lakeh M Vosoogh-Moghaddam A (2015). Health sector evolution plan in Iran; equity and sustainability concerns. Int J Health Policy Manag, 4(10):637–40. 26673172 10.15171/ijhpm.2015.160 PMC4594102

[2] Negarandeh R Dehghan-Nayeri N Ghasemi E (2015). Motivating factors among Iranian nurses. Iran J Nurs Midwifery Res, 20(4):436–41. 26257797 10.4103/1735-9066.161011 PMC4525340

[3] Salarvand S Azizimalekabadi M Jebeli AA Nazer M (2017). Challenges experienced by nurses in the implementation of a healthcare reform plan in Iran. Electron Physician, 9(4):4131–4137. 28607646 10.19082/4131 PMC5459283

[4] Erasmus E (2014). The use of street-level bureaucracy theory in health policy analysis in low-and middle-income countries: a meta-ethnographic synthesis. Health Policy Plan, 29 Suppl 3:iii70–8. 25435538 10.1093/heapol/czu112

